# Medical student interest in academic medical careers: a multi-institutional study

**DOI:** 10.1007/s40037-013-0051-6

**Published:** 2013-04-16

**Authors:** Ruth B. Greenberg, Craig H. Ziegler, Nicole J. Borges, Carol L. Elam, Terry D. Stratton, Sheila Woods

**Affiliations:** 1 University of Louisville School of Medicine, 500 South Preston Street, Louisville, KY 40202 USA; 2 Wright State University, Boonshoft School of Medicine, Dayton, OH USA; 3 University of Kentucky College of Medicine, Lexington, KY USA; 4 Medical University of South Carolina, Charleston, SC USA

**Keywords:** Medical students, Career, Academic medicine

## Abstract

Little is known about how medical students view academic medicine. This multi-institutional study explored student perceptions of this career path. During 2009–2010, third- and fourth-year students at three United States medical schools completed a 30-item online survey. In total, 239 students completed the questionnaire (37 % response rate). Significant predictors of students’ desires for academic medical careers included interest in teaching (γ = 0.74), research (γ = 0.53), interprofessional practice (γ = 0.34), administration (γ = 0.27), and community service opportunities (γ = 0.16). A positive correlation existed between accumulated debt and interest in academic medicine (γ = 0.20). Student descriptions of the least and most appealing aspects of academic medicine were classified into five categories: professional, research, personal, teaching and mentoring, and patients/patient care. Students are more likely to be interested in a career in academic medicine if they have participated in research or were influenced by a mentor. Factors that may also influence a medical student’s decision to pursue a career in academic medicine include age and debt accumulated prior to medical school. Professional aspects of academic medicine (cutting edge environment, resources) and the opportunity to teach were the most appealing aspects.

## Introduction

The 2012 Association of American Medical Colleges (AAMC) Center for Workforce Studies *Results of the 2011 medical school enrolment survey* [1] projects that first-year medical student enrolment in 2016–2017 will reach 21,376, a 29.6 % increase above first-year enrolment in 2002–2003 and will come close to reaching the 30 % target goal called for by the AAMC in 2006. As medical schools expand class sizes, they must consider how these increases will impact their ability to sustain or grow a cadre of faculty dedicated to teaching the next generation of physicians. Moreover, longstanding concerns about the future of academic medicine [2–5], the declining number of physicians entering academic medicine and faculty retention pose serious challenges to meeting the teaching needs of medical schools in an era of increased class size.

Two recent literature reviews explore influential factors in doctors’ decisions to choose an academic medical career [6, 7]. Straus et al. [6] report that academic physicians identified research experience, interest in teaching, a desire for intellectual challenge, and the influence of a mentor as factors influencing their career trajectories; the authors call for new initiatives to engage and encourage trainees to such career paths. Borges et al. [7] conclude that research-oriented programmes, gender, and mentors and role models are associated with academic medical careers; they recommend rigorous research efforts to understand better who enters academic medicine and why. Other recent works have also examined this issue of academic medical careers. Based on interviews with students, residents, and faculty at one US medical school, O’Sullivan et al. [8] identified five themes related to becoming an academic doctor: (1) early exposure to research; (2) role models and mentoring; (3) career pathways; (4) interplay of personal and social factors; and (5) career support for junior faculty. Andriole et al. [9] found that race/ethnicity, year of graduation, level of debt, expected extent (at matriculation) to which career will involve research, and type of medical school were *not associated* (emphasis added) with full-time faculty appointment. Interviews with women physicians in academic medicine to explore how, when and why they embarked on this career path revealed that these decisions were often serendipitous and not necessarily planned [10].

Although these studies provide a foundation on academic career choice, the literature, for the most part, includes retrospective studies focused on physicians. Little is known, comparatively, about medical students’ perceptions of academic medicine or what influences their interest in this career path. The purposes of this multi-institutional study were to explore medical student perceptions of and interest in academic medicine; to identify correlates associated with these interests; and to consider curricular and training implications.

## Methods

Drawing from the literature on factors that impact physician decisions about choosing a career in academic medicine, medical educators at three Midwest United States medical schools (University of Louisville, University of Kentucky and Wright State University) designed and piloted a 30-item questionnaire focused on medical careers relative to: (1) speciality choice; (2) academic medicine; (3) role models; and (4) research experience/interests. Questions included 5-point Likert-type response format items with anchors ranging from ‘Not at all interested’ to ‘Very interested’ and ‘Not at all important’ to ‘Very important’; multiple choice questions; yes/no response items; and inductive, open-ended queries on the most and least appealing aspects of academic medicine. The survey also included demographic questions on age, race, gender, and educational debt levels.

In 2009–2010, we emailed all third- and fourth-year medical students at the three schools (*N* = 650) an invitation to complete an anonymous, self-administered questionnaire hosted on a secure commercial website; we subsequently sent two email ‘reminders.’ The institutional review boards at the three participating schools approved the study protocol. We analyzed the survey responses using SPSS Version 19.0 (SPSS, 2010). We set the critical *p* value at ≤0.05, and all tests were two-tailed.

Three authors (RBG, CLE, NJB) independently coded students’ open-ended responses pertaining to the most and least appealing aspects of an academic medical career using a process adapted from Glaser and Strauss’ ‘constant comparison’ method [11]. Initially, each coder created categories or domains by noting patterns in student responses; the three coders then collectively reviewed their results, resolved differences, and refined and revised the coding domains. Next, each coder recoded the responses using the revised domains. The coders met again to review their results, resolve any remaining discrepancies, and generate sub-categories or descriptors/key terms for each domain.

## Results

The survey was completed by 239 students, representing a response rate of 37 %. The response rates for the three medical schools were 28 % (Wright State University; WSU), 34 % (University of Kentucky; UK), and 38 % (University of Louisville; UL). Of those providing demographic information, 104 (44 %) were male; 111 were female (46 %). The mean age was 26.8 years (SD 2.3); 73 % were Caucasian. See Table 1 for demographics of the sample. Comparison of population and sample demographics found the sample mean ages for each school were representative of the entire third- and fourth-year classes (UK, sample age = 26.6, class age = 27.2; UL, sample = 27.0, class = 27.3; and WSU, sample = 26.4, class = 27.5). Regarding gender, males were less likely to respond in the UK and WSU samples (UK, sample males = 45 %, class males = 61 %; UL, sample = 44 %, class = 43 %; and WSU, sample = 44 %, class = 38 %). Race also had skewed response rates (UK, sample minority status = 18 %, class minority status = 27 %; UL, sample = 21 %, class = 20 %; and WSU, sample = 11 %, class = 26 %).

**Table 1:** Demographics of the sample

Demographic	Frequency	(%)
Gender		
Female	111	46.4
Male	104	43.5
Missing	24	10.0
Race		
African-American	9	3.8
Asian/Pacific islander	17	7.1
Caucasian	173	72.4
Hispanic	2	0.8
Non-White/Non-Hispanic	1	0.4
Other	10	4.2
Missing	27	11.3
Age		
Mean ± SD	26.8 ± 2.3	

To assess the correlation of student interest in academic medicine with several practice setting-related factors we used the Goodman and Kruskal Gamma (γ). Goodman and Kruskal’s γ measures the strength of association when two variables are measured at the ordinal level with a minimal range of responses, i.e., Likert response items. Values range from −1 to +1 where −1 reflects perfect negative association and +1 reflects perfect positive association. A gamma of +1 means that a person who ranked above another person on one variable would always rank above them on the other variables as well. A value of −1 means that a person who ranked above another person on one variable would always rank below them on the other [12].

Student interest in academic medicine was significantly correlated with several practice setting-related factors: teaching opportunities (γ = 0.74), research opportunities (γ = 0.53), interprofessional practice opportunities (γ = 0.34), and administrative opportunities (γ = 0.27). Community service opportunities were also significantly related (γ = 0.16). Neither patient care opportunities nor job autonomy was associated with interest in academic medicine.

To assess the association, student interest in academic medicine was classified into two categories: (1) *low interest* (not at all, a little, and somewhat interested/important responses) and (2) *high* *interest* (pretty and very interested/important responses). Of the students who had participated in research activities, 45 % expressed high interest in academic medicine, while only 27 % who had not engaged in research activities expressed high interests (*χ* ^*2*^ = 5.54, df = 1, *p* = 0.019). Students whose speciality choice had been influenced by an academic role model also showed an increase of high interest (48 %) in academic medicine over students without an academic role model (27 %), *χ* ^*2*^ = 9.62, df = 1, *p* = 0.002.

Lastly, we found a modest but significant positive correlation between accumulated debt and interest in academic medicine (γ = 0.16, *p* = 0.016). Regarding student demographics, we found no significant relationships between gender or race and interest in an academic medicine career. However, we did find a statistically significant association for older students, who reported more interest in academic medicine than younger students [older students (≥27 years), 53 %; younger students (<27 years), 37 %, *p* = 0.035].

The two open-ended questions that asked students to write down the three factors they would find *most appealing* and *least appealing* about an academic medical career yielded 693 responses, 380 ‘most appealing’ and 313 ‘least appealing’ responses; the coding process generated five domains: 1) professional (313 responses); 2) research (61 responses); 3) personal (140 responses); 4) teaching and mentoring (123 responses); and 5) patients/patient care (56 responses). We also identified sub-categories for each domain (Table 2). Student descriptions of appealing factors in the professional domain accounted for 46 % of the most appealing responses; appealing factors in the teaching and mentoring domain accounted for 27 % of the most appealing responses. Student descriptors of the least appealing factors of a career in academic medicine focused on the professional (45 %) and personal (33 %) domains.

**Table 2:**
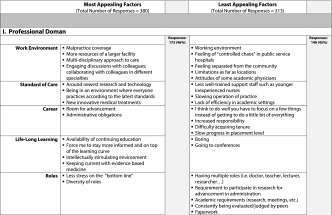

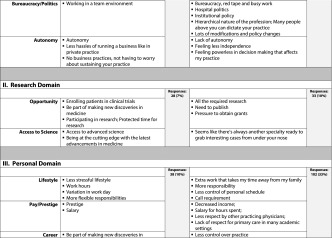
Student responses for most appealing and least appealing factors of an academic medical career with total number of responses and percentage per domain

## Discussion

This investigation explored medical student perceptions and interest in academic medical careers. Our results indicate that students were most interested in a career in academic medicine if they had participated in any research activities or were influenced by an academic role model/mentor, a finding also reported by Strauss et al. [6], Borges et al. [7, 10], and Sullivan et al. [8]. We found no relationship between gender or race and interest in a career in academic medicine, a finding previously reported by Andriole et al. [9] in their study of practising physicians. However, since diversity of teaching faculty is crucial to role modelling future generations of students, this finding suggests that medical schools should do more to target these student populations and nurture their interest in academic medicine.

We also found that older students (>27 years) were more interested in academic medical careers. It may be that older students have gleaned more research experience prior to medical school, or that they perceive academic medicine as more stimulating than private practice. When we explored debt and interest in academic medicine, we found no association between anticipated debt incurred in medical school and interest in academic medicine; however, students with greater levels of debt accumulated *prior to medical school* reported greater interest in academic medicine. This finding was surprising given the shared presumption that students with high debt levels are more likely to pursue medical practice specialities with potentially higher incomes. For these students, too, academic medicine may represent a more stable long-term income source than private practice settings or they may have acquired research exposure through costly graduate education training. We recommend replication of this area of inquiry to explore further the relationship between educational debt and career interests.

The results of the qualitative analyses (Table 2) suggest that medical students have definite views about the most and least appealing aspects of academic medicine. The medical students we surveyed listed appealing aspects of academic medicine (380) more frequently than least appealing aspects (313). Some professional aspects (e.g., resources, cutting edge environment, team approach, no business hassles) and the opportunity to teach and mentor future physicians were the most frequent responses to the open-ended question about appealing aspects of academic medicine, 173 and 103 respectively. The desire to teach and mentor the next generation of physicians was also reported by Strauss [6] as a factor related to choosing a career in academic medicine. However, students also listed some negative professional aspects of an academic medical career more frequently than any other negative aspects in response to the question about the least appealing aspects of academic medicine, comprising 140 of 313 least appealing responses (45 %), for example, the lack of control, bureaucratic structure, and separation from the community.

Our study has several study limitations. First, the response rate limits our ability to generalize our findings or conclude that they are truly representative of the student populations we surveyed. Related to this, the timing of the survey may be relevant in that we administered it just prior to Match Day, which may have dissuaded some students from responding to questions about career choice. Finally, in terms of academic medical careers, it remains unclear what effects, if any, are played by students’ impending residency choices and experiences, factors our study did not address.

Nonetheless, this study adds to our understanding of what students value in a medical career, in general, and what appeals to them about academic medicine, specifically. We felt encouraged, for example, to learn that medical students include teaching and mentoring among the most appealing aspects of academic medicine. We believe these findings have implications for career advising and curriculum revision. For example, our finding that medical students rate the teaching/mentoring aspects of academic medicine highly suggests the need for teaching electives that allow students to develop these interests [13]. Indeed, two of the study institutions have launched *medical students as teachers* electives as a possible strategy for promoting careers in academic medicine. Similarly, the positive relationship between research experience and interest in academic medicine could provide an impetus for creating additional required or elective research opportunities for medical students. We also believe that offering career workshops that openly address and discuss the most and least appealing aspects of academic medicine could also benefit medical students and medical school efforts to develop the next generation of academic physicians. Finally, career advising could include formal programmes that explore academic medicine as a career path using panels of physicians, shadowing, and other methods that expose students to academic medicine. Physicians, like other health professionals, are products of their training environments. Acknowledging and clarifying those factors that attract and detract medical students from choosing a career pathway that includes academic medicine will only bolster medical school efforts to recruit (and retain) more faculty to academic medicine.
